# Improvement of Fresh Ovine “Tuma” Cheese Quality Characteristics by Application of Oregano Essential Oils

**DOI:** 10.3390/antiox12061293

**Published:** 2023-06-17

**Authors:** Giuliana Garofalo, Marialetizia Ponte, Carlo Greco, Marcella Barbera, Michele Massimo Mammano, Giancarlo Fascella, Giuseppe Greco, Giulia Salsi, Santo Orlando, Antonio Alfonzo, Antonino Di Grigoli, Daniela Piazzese, Adriana Bonanno, Luca Settanni, Raimondo Gaglio

**Affiliations:** 1 Department of Agricultural, Food and Forest Sciences, University of Palermo, 90128 Palermo, Italy; giuliana.garofalo01@unipa.it (G.G.); marialetizia.ponte@unipa.it (M.P.); giuseppe.greco@crea.gov.it (G.G.); santo.orlando@unipa.it (S.O.); antonio.alfonzo@unipa.it (A.A.); antonino.digrigoli@unipa.it (A.D.G.); adriana.bonanno@unipa.it (A.B.); settanni.luca@unipa.it (L.S.); 2 Research Centre for Plant Protection and Certification, Council for Agricultural Research and Economics, 90011 Bagheria, Italy; carlo.greco@crea.gov.it (C.G.); massimo.mammano@crea.gov.it (M.M.M.); giancarlo.fascella@crea.gov.it (G.F.); giulia.salsi@crea.gov.it (G.S.); 3 Department of Earth and Marine Sciences, University of Palermo, 90123 Palermo, Italy; marcella.barbera@unipa.it (M.B.); daniela.piazzese@unipa.it (D.P.)

**Keywords:** oregano essential oil, lactic acid bacteria, novel fresh ovine cheese, physicochemical properties, antioxidant capacity, volatile organic compounds, sensory evaluation, dairy pathogenic bacteria

## Abstract

In the present work, oregano essential oils (OEOs) were applied to process the fresh ovine cheese “Tuma” obtained by pressed cheese technology. Cheese making trials were performed under industrial conditions using ewe’s pasteurized milk and two strains of *Lactococcus lactis* (NT1 and NT5) as fermenting agents. Two experimental cheese products (ECP) were obtained through the addition of 100 (ECP100) and 200 (ECP200) µL/L of OEO to milk, while the control cheese product (CCP) was OEO-free. Both *Lc. lactis* strains showed in vitro and in vivo ability to grow in the presence of OEOs and to dominate over indigenous milk lactic acid bacteria (LAB) resistant to pasteurization. In the presence of OEOs, the most abundant compound found in cheese was carvacrol, constituting more than 65% of the volatile fraction in both experimental products. The addition of OEOs did not influence ash, fat, or protein content, but it increased by 43% the antioxidant capacity of the experimental cheeses. ECP100 cheeses showed the best appreciation scores by the sensory panel. In order to investigate the ability OEOs to be used as a natural preservative, a test of artificial contamination was carried out, and the results showed a significant reduction of the main dairy pathogens in OEO-added cheeses.

## 1. Introduction

Cheese is an ancient, fermented food produced worldwide. Its distribution and consumption have increased over the years [1], and the cheese market is projected to reach the USD 112 billion mark by 2025 [2]. The interest of consumers toward this product is mainly due to the high content of proteins with biological value and high digestibility, minerals (e.g., calcium), vitamins, and fatty acids [3]. Such a complex nutritional composition of cheese enables the growth of the main microbial groups associated with foods, including spoilage and pathogenic species [4], endangering their stability and consumer safety [5]. Therefore, the application of chemical additives, in particular in fresh cheeses characterized by high pH and moisture, is necessary to extend their shelf life [6]. The use of chemical additives can be harmful to the consumer’s health [7]; for this reason, the dairy sector is being more and more active in proposing natural alternatives to preserve and improve the quality and safety of cheeses [8]. For this purpose, naturally synthesized substances, especially plant essential oils (EOs), are considered important to produce cheeses with long shelf lives, high nutritional values, and sensory characteristics [9].

EOs are highly hydrophobic products extracted from various parts of plants, herbs, spices, and fruits [10] added to foods for flavoring and to improve their antioxidant properties [11]. Due to their harmlessness, many EOs are included in a list (21 Code of Federal Regulations part 182) of food substances that, when used for the purposes indicated and in accordance with good manufacturing practice, are classified as GRAS (Generally Recognized as Safe) by the U.S. Food and Drug Administration [12]. EOs play an important role in food microbial control. In fact, very recently, these natural preservatives were used in food applications for their antibacterial, antifungal, antiviral, and antibiofilm formation properties [13]. Among the herbaceous aromatic plants used for the extraction of EOs, oregano assumes a role of particular interest; it is one of the most cultivated species in the Mediterranean area [14], exhibiting high antioxidant and health-promoting activities. These characteristics depend on its high content in phenolic compounds, which are mainly carvacrol and thymol [15,16]. 

Dairy products contain low concentrations of phenolic compounds [17]; thus, the addition of cheeses with plant EOs represents a novel approach to enhancing their functional properties [18]. The use of oregano essential oils (OEOs) for processing dairy foods is not new, but their application has been primarily performed with bovine milk in order to improve the microbiological and chemical properties of cheeses [19,20]. To our knowledge, no studies have been specifically performed on the evaluation of the effect of the addition of OEOs to fresh ovine cheeses. 

This study is part of a research project aimed to enlarge the ewe’s dairy product portfolio of South Italy through the use of essential oils extracted from aromatic plants of Mediterranean origin. The purpose of the present study was to evaluate, for the first time, the effect of the addition of OEOs on the microbiological, physicochemical, antioxidant, and sensory aspects of “Tuma” cheese, a Sicilian fresh-pressed cheese made from ewes’ milk characterized by a typical cylindrical shape with a uniform structure and a white or ivory white color [21].

Cheese making trials were performed at an industrial scale using *Lactococcus lactis* starter cultures and using OEOs as natural antimicrobial substances. For this purpose, a scale-up approach was followed to test, firstly, the antibacterial activity of OEOs against the main dairy pathogenic bacteria in vitro; second, after efficacy evaluation, the same components were applied in vivo. This study also aimed to evaluate the chemical composition of the OEOs, monitor starter lactic acid bacteria (LAB) during cheese production, and evaluate the physicochemical, antioxidant, volatile organic compounds, and sensory traits of the final cheeses.

## 2. Materials and Methods

### 2.1. Oregano Essential Oil Extraction and Gas Chromatography Analysis

The plants of *Origanum vulgare* ssp. *viridulum* × *Origanum vulgare* ssp. *hirtum* were provided by a farm located in Agrigento (37°27’28’’ N, 13°36’01’’ E). After harvesting, the plants were air dried and transferred into plastic bags at the laboratories of the Research Centre for Plant Protection and Certification (Bagheria, Italy). EOs were extracted from the leaves and flowers using a distiller in a current of steam of 12 L volume (Spring Extractor, Albrigi Luigi, Verona, Italy). After extraction, OEOs were transferred to 25 mL amber glass bottles with a screw cap (Laboindustria, Arzergrande, Italy) and stored at 4 °C.

Volatile Organic Components (VOC) of oregano essential oils were analysed by solid-phase microextraction (SPME) GC–MS after dilution with hexane (1:100). The SPME fiber (DVB/CAR/PDMS, 50 mm, Supelco, Bellefonte, PA, USA) was exposed to the diluted oils under stirring at 60 °C. After an extraction time of 5 min, fiber was inserted in a GC splitless injector, and volatile organic components were desorbed for 1 min at 250 °C. Chromatographic separation was performed using a DB-624 capillary column (Agilent Technologies, Santa Clara, CA, USA, 60 m, 0.25 mm, 1.40 µm). The oven temperature program was set with a 5 min isotherm at 40 °C followed by a linear temperature increase of 5 °C every minute up to 200 °C, where it was held for 2 min and the helium carrier gas was set at 1 mL/min. The interface temperature was fitted at 230 °C and mass spectra were recorded in the range of m/z 40–400 amu under full-scan acquisition mode. Single volatile organic compounds were identified by comparing each MS spectra with the commercial library NIST05. Results were obtained from three replicates and reported as percentages relative to the significant peak.

### 2.2. Bacterial Strains, Milk Starter Culture Preparation, and Culture Conditions

In order to evaluate the antibacterial properties of OEOs, four bacterial strains belonging to the American Type Culture Collection (ATCC) were used as indicators of microorganisms (sensitive to antimicrobial compounds). In particular, two gram-positive (*Listeria monocytogenes* ATCC19114 and *Staphylococcus aureus* ATCC33862) and two gram-negative (*Escherichia coli* ATCC25922 and *Salmonella* Enteritidis ATCC13076) bacteria were chosen as representatives of the main dairy bacterial pathogens. These bacteria were reactivated in Brain Heart Infusion (BHI) broth (Condalab, Madrid, Spain) and incubated at 37 °C for 24 h.

Milk starter cultures (MSC) were developed with two strains of *Lc. lactis* (NT1 and NT5) belonging to the culture collection of the Department of Agricultural, Food, and Forest Sciences (University of Palermo, Italy). These strains were previously isolated from LYOBAC-D NT freeze-dried starter preparation (Alce International s.r.l., Quistello, Italy) [22]. *Lc. lactis* strains were cultivated in M17 broth (Oxoid, Hampshire, UK) at 30 °C for 24 h and centrifuged at 10,000× *g* for 5 min. The cells were then washed twice in Ringer’s solution (Oxoid) and re-suspended in the same solution. The washed cells of *Lc. lactis* were inoculated (1%, *v*/*v*) into ovine whole fat UHT milk (Leeb Vital, Wartberg an der Krems, Austria) and incubated at 30 °C for 24 h [23]. MSC containing the multi-strain culture at about 10^9^ CFU/mL, as verified by plate count, was then used for cheese production.

### 2.3. In Vitro Antibacterial Activity of Oregano Essential Oil

The antibacterial activity of the OEOs was tested in vitro against a cell density of approximately 10^7^ CFU/mL of the four pathogenic bacterial strains (*E. coli*, *L. monocytogenes*, *S.* Enteritidis, and *St. aureus*) and the two LAB (*Lc. lactis*) in BHI or M17 soft agar (0.7% *w*/*v*) by the paper disc diffusion method, as indicated by Gaglio et al. [24]. Streptomycin at 10% (*w*/*v*) was used as a positive control, while sterile water was used as a negative control [25]. After incubation at 37 °C for 24 h, inhibitory activity was assessed and considered positive only if a clear area around the paper discs was present. The test was carried out in triplicate.

Once scored positive, the antibacterial activity against the four pathogenic bacteria was quantified as Minimum Inhibitory Concentration (MIC) following the methodology reported by Militello et al. [26]. Briefly, the OEOs were serially 2-fold diluted in acetone (Carlo Erba Reagents, Rodano, Italy), added to Brain Heart Infusion (BHI) broth (Condalab, Madrid, Spain), and tested by employing the strains at approximately 10^6^ colony forming units (CFU)/mL. The test was carried out in triplicate.

### 2.4. Description of Dairy Plant

**Figure 1:**
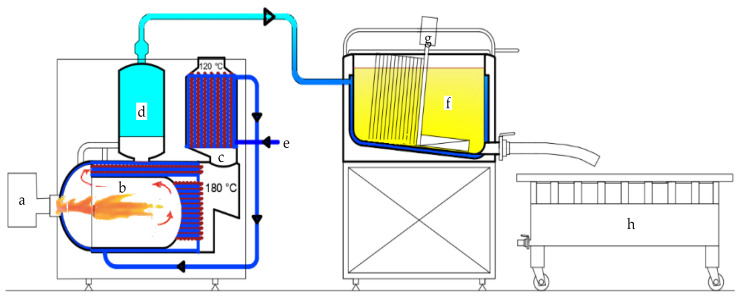
Scheme of dairy plant. (**a**) diesel burner; (**b**) furnace (first and second stage of smoke circuits); (**c**) third stage of smoke circuits; (**d**) steam accumulator with dispensing valve; (**e**) water inlet; (**f**) multipurpose coagulation vats; (**g**) gearmotor for mechanically cutting curd; (**h**) perforated steel table.

Cheese was produced on an industrial scale at the dairy factory Azienda Agricola Giuseppe Basile in Ventimiglia di Sicilia (Palermo, Italy). The dairy plant (Figure 1) used for cheese production is a multi-purpose system with a working capacity of 200 L (Sfoggia & C. SAS, Montebelluna, Italy).

The plant is equipped with a high-efficiency wet-bottom condensing steam generator (Figure 1a–e) with flame reversal, and it is fueled by diesel (TFRE series, Manara Roberto S.r.l Company, Fontevivo, Italy). The generator is characterized by a steam production of 150 kg/h, a stamp pressure of 5 ÷ 12 bar, a potential yield of 90,000 kcal/h, and horizontal smoke tubes. There is a fully inspectable front cavity door with water recirculation from the generator. There are three passes of smoke (the first and second in the hearth, the third in the pipes) to increase efficiency. The feed water flows against the current, raising the temperature by over 40 °C and, consequently, lowering the flue gas temperature to 120 °C, thus obtaining an efficiency of 95%. It is completed with the automatic drain in electro-pneumatic stainless steel adjustable by PLC with functions also in manual mode. The tank used for milk pasteurization and coagulation (Figure 1f) is double-bottomed, steam heated, and equipped with a gearmotor for mechanically cutting curd (Figure 1g). It is connected to two probes, which transmit into an electrical panel the temperature of the milk, curd, and heating water. The bottom of the tank is slightly inclined and has a drain valve, located in the lower part, through which it is possible to drain the curd and whey. A perforated steel table (Figure 1h) is used for curd molding. 

### 2.5. Cheese Production and Sample Collection

**Figure 2:**
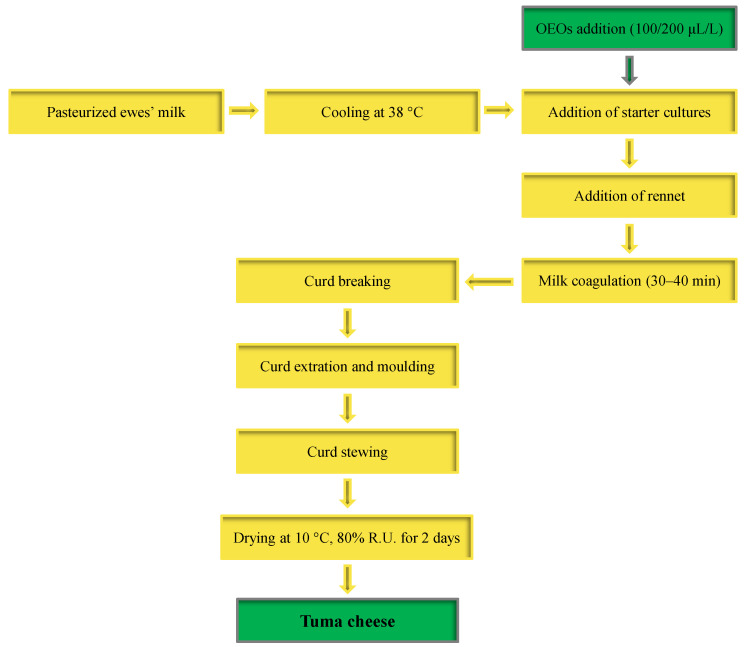
Flow diagram of Tuma cheese production. Abbreviation: OEOs, oregano essential oils.

Pasteurized ewe’s milk (60 °C for 30 min) from crossbreeds between “Valle del Belice” × “Sarda” sheep was used as the raw material. Cheese making trials were performed by applying Tuma pressed cheese technology (Figure 2). 

Three cheese products were obtained using MSC as a fermenting agent: CCP, a control cheese product prepared from pasteurized ewe’s milk; ECP100, an experimental cheese product prepared from pasteurized ewe’s milk enriched with 100 µL/L of OEOs; and ECP200, an experimental cheese product prepared from pasteurized ewe’s milk enriched with 200 µL/L of OEOs. Each production was performed in a stainless steel vat previously sanitized with a DTH101 isopropyl solution (Trezzo sull’Adda, Italy) with 200 L of pasteurized whole ewe’s milk. After cooling at 40 °C, the milk of the CCP and ECP trials was inoculated with the MSC at a concentration of approximately 10^7^ CFU/mL. Before liquid rennet (Micromilk Srl, Cremosano, Italy) was added (60 mL), the milk used for the ECP productions was inoculated with 100 and 200 µL/L of OEO, respectively. After curdling, the curd was cut until attaining the dimension of small rice-size grains, which were hand pressed into 1 kg cylindrical perforated plastic molds, kept at 40 °C for 50 min (the so-called stewing step), and then dried for 2 days at 10 °C. All cheese productions were replicated for two consecutive weeks. Samples of raw milk, pasteurized milk, inoculated milk after the addition of MSC and OEOs, whey, curds, and cheeses after two days following production were collected for analyses.

### 2.6. Microbiological Analyses

First, 1 mL of the liquid (milk and whey) samples was directly serially diluted in Ringer’s solution (Oxoid), while 10 g of the solid (curd and cheese) samples was first homogenized in 90 mL of sodium citrate (2% *w*/*v*) solution in the Bag-Mixer 400 stomacher (Interscience, Saint Nom, France) at the maximum speed for 1 min and then serially diluted in Ringer’s solution (Oxoid). Appropriate dilutions of raw milk and pasteurized milk were plated on agar media to allow the development of: total mesophilic microorganisms (TMM) spread on Skim Milk Agar (SMA) (Microbiol Diagnostici, Cagliari, Italy) and incubated for 72 h at 30 °C; mesophilic lactic acid bacteria (LAB) cocci poured in Medium 17 (M17) agar (Oxoid) incubated for 48 h at 30 °C; mesophilic LAB rods poured in de Man-Rogosa-Sharpe (MRS) agar (Condalab), adjusted to pH 5.4 with 5 Mol lactic acid, incubated for 48 h at 30 °C; enterococci spread on kanamycin Esculin Azide (KAA) agar (Biotec, Grosseto, Italy) incubated for 24 h at 37 °C; *Pseudomonas* spp. spread on *Pseudomonas* Agar Base (PAB) (Condalab) incubated at 22 °C for 72 h; members of the Enterobacteriaceae family poured in Violet Red Bile Glucose Agar (VRBGA) (Biolife Italiana, Monza, Italy) incubated for 24 h at 37 °C; *E. coli* spread on Coliforms Chromogenic Medium (CHROM) agar (Condalab); *L. monocytogenes* spread on Agar *Listeria* to Ottaviani and Agosti (ALOA) added with ALOA enrichment-selective supplement (Biolife Italiana) incubated for 24 h at 37 °C; *Salmonella* spp. spread on Xylose Lysine Deoxycholate (XLD) agar (Liofilchem, Roseto degli Abruzzi, Italy) incubated for 24 h at 37 °C; and coagulase-positive staphylococci (CPS) spread on Baird-Parker (BP) agar added with enrichment-selective supplement (Oxoid) incubated for 48 h at 37 °C. All plate counts were incubated aerobically, except those used for growing LAB, which were incubated anaerobically using the AnaeroGen AN25 system (Oxoid).

The appropriate dilutions of inoculated milk, curd, and whey and cheese samples were analyzed for TMM and mesophilic coccus LAB exclusively on M17 agar (Biotec). Plate counts were carried out in triplicate.

### 2.7. Monitoring of Starter Cultures and Identification of the Thermoduric Milk LAB

In order to monitor the dominance of *Lc. lactis* (NT1 and NT5) inoculated as a starter culture over thermoduric LAB, all presumptive LAB collected during cheese making were analysed by the randomly amplified polymorphic DNA (RAPD)-PCR technique, as reported by Gaglio et al. [27]. Briefly, the analysis was performed using the single primers M13 (5′-GAGGGTGGCGGTTCT-3′), AB111 (5′-GTAGACCCGT-3′), and AB106 (5′-TGCTCTGCCC-3′) in a 25 μL reaction volume. The PCR program applied to all primers comprised an initial template denaturation step for 2 min at 94 °C, followed by 40 cycles of denaturation for 1 min at 94 °C, annealing for 20 s at 40 °C, extension for 2 min at 72 °C, and a final extension at 72 °C for 10 min. Amplifications were performed by means of Swift™ MaxPro Thermal Cycler (Esco Technologies Inc., Oak Ridge, NJ, USA). The obtained polymorphic profiles of the axenic cultures of *Lc. lactis* strains were compared to those of the LAB colonies developed on agar media using the software Gelcompare II version 6.5 (Applied-Maths, Sint-Martens-Latem, Belgium). All different LAB strains isolated from pasteurized milk before MSC addition were subjected to 16S rRNA gene sequencing following the procedures applied by Weisburg et al. [28]. The resulting DNA fragments of about 1600 bp were purified by the ExoSAP-IT™ Express PCR Product Cleanup Reagent (Thermo Fisher Scientific, Waltham, MA, USA) and sequenced at BMR Genomics (Padova, Italy). The unequivocal identities of the sequences were determined by comparison with available data in two distinct databases, as reported by [29].

### 2.8. Physicochemical Analysis of Tuma Cheeses

The assessment of cheese color was performed in duplicate by a Minolta Chroma Meter CR-300 (Minolta, Osaka, Japan) measuring the values of lightness (L* = 0–100, from black to white), redness (a* = −a/+a, from green to red), and yellowness (b* = −b/+b, from blue to yellow), according to the CIE L*a*b* system [30]. The evaluation of maximum resistance to compression (compressive stress, N/mm^2^) was performed by measuring the hardness of cheese samples (2 × 2 × 2 cm) maintained at 22 °C (room temperature) using an Instron 5564 tester (Instron, Trezzano sul Naviglio, Milan, Italy). The water activity (a_w_) of different samples was measured with the HygroPalm portable water activity meter (Rotronic, Bassersdorf, Germany), according to ISO 21807 [31].

Lyophilized cheese samples were analysed for dry matter (DM), protein (N × 6.38), fat, and ash content in accordance with International Dairy Federation (IDF) standards [32,33,34]. Extracts of lyophilized cheese samples were prepared according to the method of Rashidinejad et al. [35] with minor changes. Briefly, a milled cheese sample (0.5 g) was dissolved in methanol 95% aqueous solution (25 mL) added with 1% HCl. The suspension was mixed by vortex for 30 s and then maintained at 40 °C in an ultrasonic water bath (LBS1 Sonicator; Falc Instruments, Treviglio, Italy) for 30 min, during which time it was mixed for 5 s every 10 min. Then, the suspension was cooled, filtered with linen a cloth, centrifuged at 7000 rpm at 9 °C for 10 min, and kept at −18 °C until analysis.

### 2.9. Antioxidant Capacity of Tuma Cheeses

Analyses for antioxidant properties of cheeses were performed in duplicate on extracted samples by TEAC (Trolox equivalent antioxidant capacity) assay. 

The total antioxidant capacity (TEAC) in extracted cheese samples was measured by TEAC assay as Trolox equivalent according to a published procedure [33], as described by Bonanno et al. [36] (with some modifications). TEAC is a decolorization assay by which samples are evaluated for their radical scavenging ability using the ABTS radical cation (ABTS^•+^) and Trolox as standard [37]. To obtained the ABTS radical cation, equal volumes of a 14 mM ABTS aqueous solution and 4.9 mM potassium persulphate were mixed and incubated in the dark for 16 h at room temperature. To perform the assay, the ABTS radical cation solution was diluted in 5 mM phosphate buffered saline (PBS, pH 7.40) until obtaining an absorbance of 0.795 (±0.020) at 734 nm by the Hach DR/4000 U spectrophotometer. The mixture of 150 µL of PBS with 2850 µL of a diluted ABTS radical cation solution was placed in a cuvette, and its absorbance was recorded at 734 nm immediately and after incubation for 6 min at 30 °C. At the same way, 150 µL of extracted samples was mixed with 2850 µL in the same diluted solution of ABTS radical cation, and their absorbance was read at 734 nm after a 6 min incubation at 30 °C; using the read absorbance, the percentage decrease of the absorbance due to decolorization was calculated in comparison with the absorbance obtained with PBS. Solutions of Trolox in PBS (0–2.5 mM) were used to construct a calibration curve (R^2^ = 0.99), with results expressed as mmol Trolox/kg DM.

The stability and susceptibility of cheese fat to oxidation were estimated by determining in duplicate the POV (peroxide value, mEq O_2_/kg fat) expressing the primary lipid oxidation [38], and TBARs (thiobarbituric acid-reactive substances, μg of malonylaldehyde (MDA)/kg DM) as products of secondary lipid oxidation, as described by Tarladgis et al. [39] and slightly modified by Mele et al. [40].

Briefly, for TBARs analysis, 2 g of lyophilized cheese was mixed with 8 mL of aqueous solution of phosphate buffer (pH 7) and vortexed. Then, the sample was added to 2 mL of 30% (*v*/*v*) aqueous solution of trichloroacetic acid and vortexed for 5 s and filtered with Whatman filter paper No. 1. Then, 5 mL of filtrate was added with 0.02 M thiobarbituric acid aqueous solution and placed for 20 min in a hot water bath at 90 °C and then refrigerated. After centrifugation (4500 RPM for 5 min), the reading of the supernatant absorbance was performed at 530 nm by the Hach DR/4000 U spectrophotometer. Solutions of 1,1,3,3-tetramethoxypropane at concentrations between 0.016 and 0.165 µg/mL were read to construct the calibration curve (R^2^ = 0.99).

### 2.10. Volatile Organic Compounds Emitted from Tuma Cheeses

Volatile organic compounds (VOCs) of cheese samples were extracted by SPME and identified using the GC–MS technique. Two grams of both control and OEOs-enriched cheese samples were finely chopped and placed in a glass vial for the headspace solid-phase microextraction. Both extraction and desorption of the analytes from the SPME fiber were carried out by applying the same procedure reported above for the determination of the aromatic profile of oregano essential oil (Section 2.1). Furthermore, chromatographic and mass spectrometry instrumental conditions set up for the analysis of the OEOs’ volatile profile were also applied to obtain the mass spectra of the volatile compounds emitted by the cheeses.

### 2.11. Sensory Evaluation of Tuma Cheeses

The sensory properties of CCP and ECP cheeses were evaluated following the ISO [41] indications by a descriptive panel of 16 evaluators, including 7 women and 9 men aged between 22 and 64 years old. The evaluators were trained in order to recognize the specific attributes for Tuma cheese and were asked to score 22 descriptors grouped into aspect, aroma, taste, and texture categories, as reported by Ashkezary et al. [42]. All sensory attributes were evaluated using a hedonic scale from 1 to 9 (1 = extremely low; 9 = extremely high).

### 2.12. In Vivo Antibacterial Effect of Oregano Essential Oil

To thoroughly investigate the antibacterial activity of OEOs during the production of Tuma cheese, tests of artificial contamination were carried out. Three additional cheese making trials were performed under controlled laboratory conditions, including one control production without the addition of OEOs and experimental productions obtained by the addition of 100 and 200 μL/L of OEOs to milk. Before OEOs were added, the milk of all trials was inoculated with 10^7^ CFU/mL of the starter cultures (*Lc. lactis* NT1 and NT5) and 10^4^ CFU/mL of pathogenic bacteria (*E. coli* ATCC 25922, *L. monocytogenes* ATCC 19114, *S.* Enteritidis ATCC13076, and *St. aureus* ATCC 33862) to simulate a massive contamination. Each trial was carried out with 20 L of pasteurized whole ewe’s milk following the protocol of production reported above (Section 2.4). Samples of pasteurized ewe’s milk, inoculated milk after the addition of MSC, pathogenic bacteria and OEOs, curds, and cheeses after two days from ripening were subjected to microbiological analysis.

### 2.13. Statistical Analyses

Microbiological and VOC data were subjected to One-Way Analysis of Variance (ANOVA) using XLStat software version 7.5.2 for Excel (Addinsoft, New York, NY, USA). Differences between means were determined by Tukey’s test at *p* ≤ 0.05.

The physicochemical data were statistically analyzed using the MIXED procedure in SAS 9.2 software (SAS Institute Inc., Campus Drive Cary, NC, USA). In the mixed model, cheese production (3 levels: CCCP, ECCPO100, ECCPO200) represented the fixed factor, and cheese making (2 levels as replicates) was the random factor used as the error term. When the effect of production was significant (*p* ≤ 0.05), the averages were compared using *p*-values adjusted according to multiple comparison Tukey–Kramer.

## 3. Results and Discussion

### 3.1. Chemical Composition of Oregano Essential Oil

**Figure 3:**
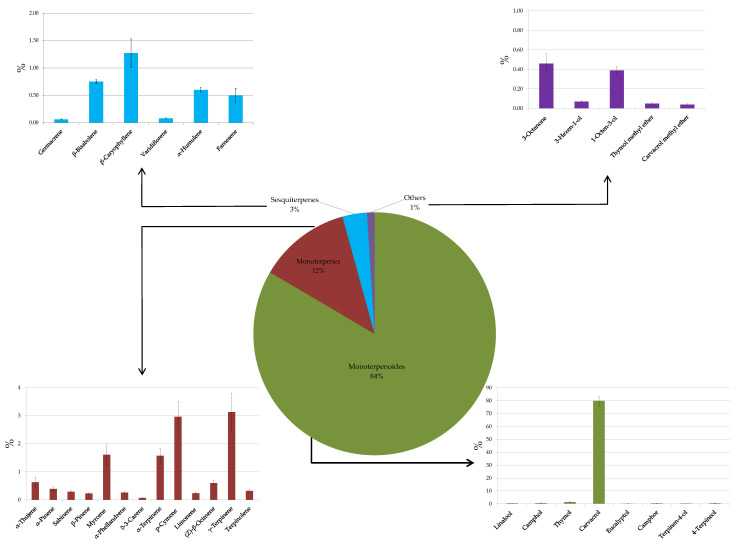
Volatile organic compounds emitted from oregano essential oil. Results indicate mean percentage values ± standard deviation (S.D.) of three measurements and are expressed as relative peak areas (peak area of each compound/total area of the significant peaks to all samples) × l00.

Figure 3 reports the graphical representation of the volatile profile of OEOs. Globally, thirty-two volatile compounds belonging to six phytochemical groups (monoterpenes, monoterpenoids, sesquiterpenes, ethers, ketones, and alcohols) were found.

Most of the compounds identified belong to the group of monoterpenes; among these, the most abundant chemicals were p-cymene, γ-terpinene, and myrcene. However, the most abundant volatile compound detected was carvacrol (79.9 ± 3.5%), determining the monoterpenoids groups as the most abundant class of VOCs in OEOs analysed in this study. Carvacrol, p-cymene, and γ-terpinene are also the main compounds detected in other oregano plants grown in the Mediterranean area [43,44,45]. Although the volatile composition of OEOs may be affected by many factors (environmental conditions, season of collection, age of plants, and geographical origin) [44,46,47], several studies have reported the possibility of classifying oregano chemotypes according to their essential oil composition in carvacrol type, thymol type, and both of them (carvacrol and thymol in almost equal amounts) [43,46,48]. According to Fleisher and Sneer’s [48] classification, our results show that because of the higher content of carvacrol (~80%), the OEOs studied corresponded to the carvacrol oregano type, thus determining the smell and use of the condiment oregano. Besides characterizing OEOs’ aroma, carvacrol exhibits a plethora of bioactivities, including antioxidative properties, inhibition of antibiotic-resistant bacteria, inhibition of microbial and fungal toxins, and anti-carcinogenic activity [49,50,51,52]. These aspects highlight the potential of using OEOs belonging to the carvacrol oregano type as a multifunctional food ingredient.

### 3.2. Antibacterial Activity of Oregano Essential Oil

The antibacterial activity of OEOs against the four main dairy pathogens (*E. coli*, *L. monocytogenes*, *S.* Enteritidis, and *St. aureus*) responsible for foodborne diseases associated with cheese consumption [53] and the two LAB (*Lc. lactis*) commonly used as fermenting agents in dairy production [54] is shown in Table 1.

The high sensitivity of human pathogens to EOs extracted from different varieties of oregano cultivated in Sicily is well known [55]. However, OEOs tested in this study showed very high antibacterial activity against all pathogenic strains with a diameter of the inhibition area around the paper disc in the range 35.8–42.5 mm. Considering the strong activity shown by OEOs, it was also characterized in terms of an MIC that represents the lowest concentration of an active compound able to inhibit microbial growth [56]. MIC confirmed the strong activity of OEOs tested in this study with values of 2.50 µL/mL against *L. monocytogenes*, 1.25 µL/mL against *S.* Enteritidis and *St. aureus*, and 0.625 µL/mL against *E. coli*. These results suggest that this natural product has great potential in food preservation to ensure for consumers a safe food supply. Interestingly, the development of the lactococci selected as starter cultures was not inhibited by OEOs, indicating their harmlessness against LAB, a basic condition for their application in cheese making. The resistance of LAB to the OEOs’ components may be explained by the fact that bacterial susceptibility to antimicrobial agents is strain dependent [57].

### 3.3. Evolution of Microbial Populations during Cheese Making

The results of the plate counts carried out in ewes’ milk before and after pasteurization are reported in Table 2. 

**Figure 4:**
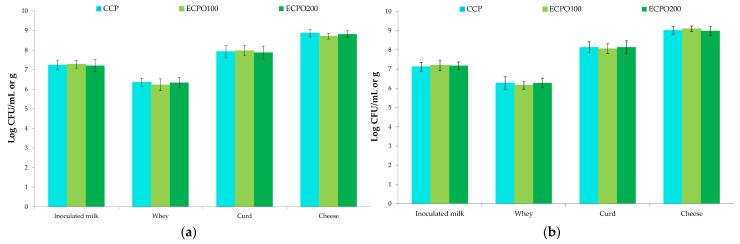
Growth of starter cultures during Tuma cheese productions. (**a**) total mesophilic microorganisms; (**b**) *Lactococcus lactis*. Units are Log CFU/mL for milk and whey samples and Log CFU/g for curd and cheese samples. Abbreviations: CCP, control cheese product inoculated with the milk starter cultures (MSC); ECPO100, experimental cheese product inoculated with MSC + 100 μL/L of oregano essential oils (OEOs); ECPO200, experimental cheese product inoculated with MSC + 200 μL/L of OEOs.

Raw ewes’ milk hosted levels of TMM, mesophilic coccus, and rod LAB of 6.02, 5.71, and 4.27 Log CFU/mL, respectively. Similar results were previously reported by Guarcello et al. [58] and by Gaglio et al. [59] in raw milk used for the production of two traditional Sicilian cheeses; specifically, PDO Pecorino Siciliano and PDO Vastedda della valle del Belìce. After the application of the pasteurization treatment, the levels of TMM, coccus, and rod LAB decreased by about three Log cycles, as previously observed by Barbaccia et al. [22,60] for raw and pasteurized ewe’s milk used for ovine pressed and stretched cheese productions. These results confirmed the ability of LAB to survive during thermal pasteurization [61]. *E. coli* and CPS, analysed through microbiological process hygiene criteria [62], were found at low levels (2.79 and 2.42 Log CFU/mL, respectively) in raw ewes’ milk and decreased below the detection limit in pasteurized milk. Regarding *L. monocytogenes* and *Salmonella* spp., which are associated with the microbiological food safety criteria [62], they were never detected in either of the matrices. The microbiological counts of inoculated milk after the addition of MSC and OEOs and whey, curd, and cheese samples included only TMM (Figure 4a) and *Lc. lactis* (Figure 4b) to evaluate the ability of the starter LAB to act as a fermenting agent in the presence of OEOs.

According to Tukey’s test, no statistically significant differences were found for the levels of TMM and *Lc. lactis* in all the samples analysed. In particular, these microorganisms were found in inoculated milk used for CCP and ECP productions at almost the same levels inoculated, confirming that *Lc. lactis* inoculums occurred at 10^7^ CFU/mL. The analysis of whey at the time of curd separation showed a decrease of about one Log cycle for TMM and *Lc. lactis* that were present at levels of about 6 Log CFU/mL. Settanni et al. [63] observed a similar behaviour by analysing cows’ milk and whey samples during the production of Caciocavallo-type cheese. Control and experimental curds showed values of TMM and *Lc. lactis* of about 10^8^ CFU/g, and their levels reached values of about 9 Log CFU/g in the final cheeses, showing clearly that the addition of OEOs did not interfere with *Lc. lactis* development. Marcial et al. [64] observed a similar behavior in cows’ milk inoculated with OEOs used for the production of Argentinean fresh bovine cheese.

### 3.4. Composition of Thermoduric LAB Populations

**Figure 5:**
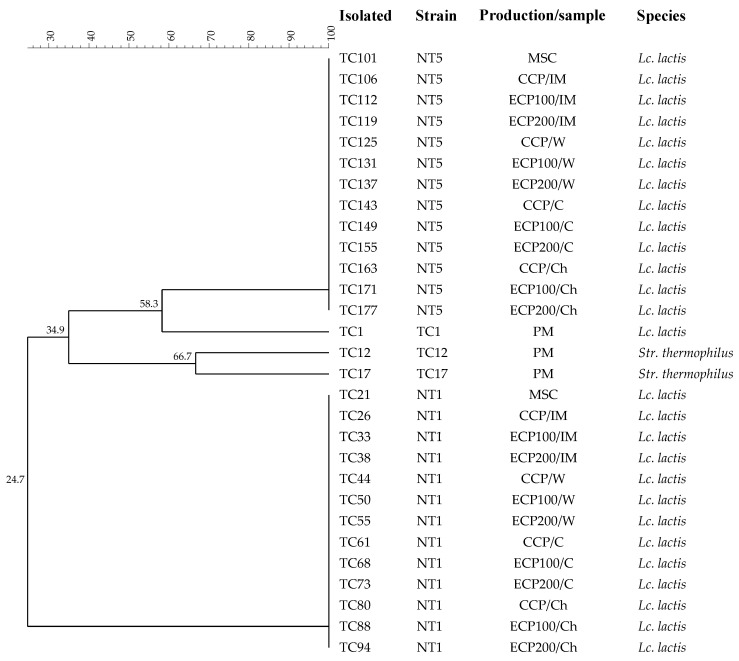
Dendrogram obtained from combined RAPD-PCR patterns of LAB strains isolated from pasteurized ewe’s milk to Tuma cheeses. Abbreviations: CCP, control cheese product inoculated with the milk starter cultures (MSC); ECPO100, experimental cheese product inoculated with MSC + 100 μL/L of oregano essential oils (OEOs); ECPO200, experimental cheese product inoculated with MSC + 200 μL/L of OEOs; PM, pasteurized milk; IM, inoculated milk; W, whey; C, curd; Ch, cheese; *Lc.*, *Lactococcus*; *Str.*, *Streptococcus*.

One hundred and eighty-two colonies of LAB (gram-positive and catalase-negative) were isolated from all samples collected during cheese production, from pasteurized ewe’s milk before MSC addition to processed cheeses. All isolates were subjected to RAPD analysis, a technique commonly used for strain typing and to monitor the starter LAB deliberately added [65]. The dendrogram reported in Figure 5 shows only 29 strains; this is because the rest of the isolates were characterized by polymorphic profiles already included in the dendrogram.

Two major RAPD clusters were identified. Each cluster included one of the *Lc. lactis* strains used for MSC preparation. In particular, *Lc. lactis* (NT1 and NT5) was detected in all samples collected from the control and experimental products. Three different strains isolated from pasteurized ewe’s milk were identified as *Lc. lactis* subsp. *lactis* (Ac. No. OQ675106) and *Str. thermophilus* (Ac. No. OQ675104-OQ675105). These species are part of the typical starter LAB cultures, which are able to generate a high amount of lactic acid during the very first steps of cheese production through the fermentation of lactose [66]. Although they are commonly part of raw milk microbiota [67], their ability to survive the pasteurization process is well known [68,69]. However, raw milk LAB strains were not found at dominant levels in any of the samples analysed after the addition of MSC, evidencing the ability of the starter cultures to dominate over the indigenous milk LAB that survived to the pasteurization treatment. These results confirmed those obtained by microbial counts that excluded any negative influence of OEO during the fermentation process.

### 3.5. Physicochemical Characterisation of Tuma Cheeses

The physicochemical traits of the cheeses are reported in Table 3. The chemical composition of cheese in terms of ash, protein, and fat was not affected by the addition of OEO. Additionally, chemical components of the processed cheeses were in the ranges registered in other investigations [18,70,71].

The lowest hardness, expressed as resistance to compression, was found in the experimental production cheeses (ECPO100 and ECPO200), which reflects the lower consistency of their paste, justified by their higher humidity and then by their lower DM percentage.

Statistically significant differences were obtained for all cheese color indexes. Indeed, experimental products at both OEO levels (ECPO100 and ECPO200) showed higher values of lightness (L*) and redness (a*) and a corresponding lower yellow index (b*) compared to CCP cheeses. These results were expected considering the dark color of the extract attributed to the chlorophyll content in oregano. Furthermore, Boroski et al. [72], who added OEOs to milk, found that the color varied more consistently at increasing OEO concentrations. However, the color indexes recorded in CCP cheeses were within the ranges observed for this type of cheese [18,36].

### 3.6. Antioxidant Capacity of Tuma Cheeses

The results of the antioxidant capacity of the cheeses analysed are reported in Table 3. The antioxidant capacity of OEO-added cheeses, expressed as TEAC, was higher than that registered for CCP; in particular, the highest antioxidant capacity was observed for the cheeses produced with the addition of 200 µL/L of OEOs. This result confirmed the well-known antioxidant properties of oregano, mainly due to the presence of relevant contents in phenolic compounds, such as carvacrol and thymol [15,16]. For this reason, OEOs are commonly used in food production to prolong shelf life by preventing oxidation and to preserve health properties.

Cheese fat stability to oxidation is expressed by POV and TBARs, representing, respectively, the indexes of primary and secondary lipid oxidation. The inclusion of OEOs was able to increase the antioxidant capacity of the cheeses, while it was not able to avoid an early fat oxidation. Indeed, the POV level was lower in 48 h control cheeses, whereas TBARs increased with the OEOs’ inclusion. Similar results were obtained by Busetta et al. [69], who produced cheeses with EOs from citrus fruits. Based on these results, further investigations are necessary to explore the antioxidant potential of OEOs in preserving the oxidative stability of cheese fat during a prolonged storage time.

### 3.7. Volatile Organic Compounds Emitted from Tuma Cheeses

Table 4 shows the VOC profiles generated from cheese samples with and without OEOs’ addition. Twenty-one compounds were detected in control cheeses: seven acids, five ketones, four aldehydes, and four alcohols.

The main class found in cheese samples was free fatty acids (FFA). Hexanoic acid showed the highest values, followed by butanoic and acetic acid. Butanoic and hexanoic acids may generally result from the lipolysis of milk fat due to the action of the lamb rennet used for curdling, and also, in part, due to the activity of raw milk lipoprotein lipase [73,74]. Acetic acid can originate from different processes, including the oxidation of lactose by lactic acid bacteria under anaerobic conditions and carbohydrate catabolism by lactic acid bacteria [75]. Other odor-active compounds, such as alcohols (1-butanol-3-methyl (isoamyl alcohol) and aldehydes (hexenal and heptanal), were also revealed. Globally, the volatile composition detected in control cheeses reflects the volatile profile of cheeses produced from sheep’s milk, as observed in many studies [18,76]. SPME-GC–MS analysis clearly showed the effect of OEOs’ addition, as both experimental products (ECPO100 and ECPO200) were characterized by components characteristic of the control cheese, such as hexanoic, butyric, and acetic acids and hexenal, heptanal, and isoamyl alcohol. However, the major volatile fraction was constituted by the typical compounds detected in OEOs, including, first of all, carvacrol. Furthermore, compounds belonging to the monoterpene hydrocarbons class, such as γ-terpinene, myrcene, β-pinene, p-cymene, and α-terpinene, and oxygenated monoterpenes, such as linalool, thymol, and β-caryophyllene, were also detected. Our results showed that independently of the amount of OEOs added, generally, no significant differences were found for VOCs emitted from cheese, but the percentage of carvacrol significantly increased. A similar effect was observed by Busetta et al. [69] on the VOC profiles of cheeses processed with different concentrations of citrus EOs. These results revealed that a small addition of OEOs (100 µL/L of OEO) significantly influenced the cheese’s flavor profile, demonstrating that OEO compounds are easily transferred to cheese.

### 3.8. Sensory Aspects of Cheeses

**Figure 6:**
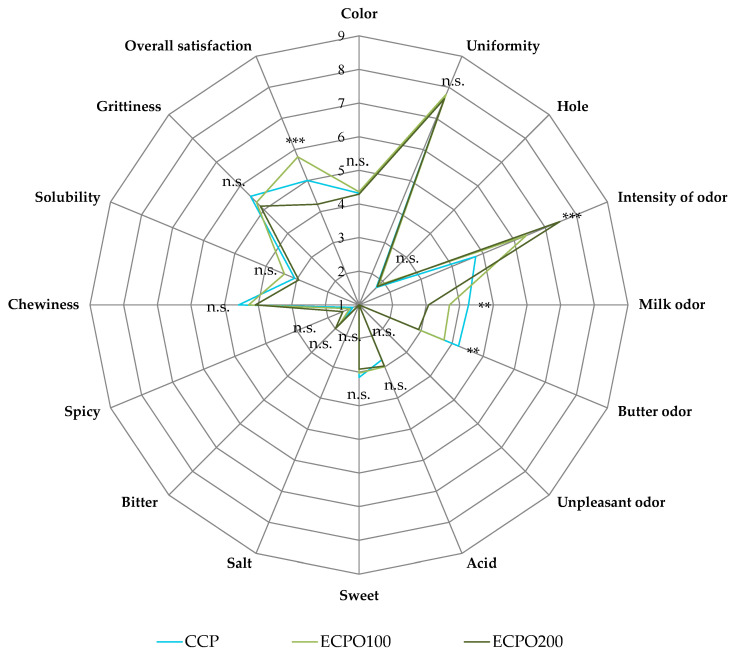
Spider diagram of descriptive sensory analysis of Tuma cheeses. Abbreviations: CCP, control cheese product inoculated with the milk starter cultures (MSC); ECPO100, experimental cheese product inoculated with MSC + 100 μL/L of oregano essential oils (OEOs); ECPO200, experimental cheese product inoculated with MSC + 200 μL/L of OEOs. ** *p* < 0.01; *** *p* < 0.001; n.s., not significant.

Figure 6 reports the spider plot of the sensory attributes evaluated on control and experimental cheeses after 2 d of ripening. This approach makes it feasible to predict the consumer’s attitude toward a new food product before its production at an industrial level and its introduction into the marketplace [77]. In this study, the addition of OEOs did not particularly affect the sensory attributes of Tuma cheeses. Except for the intensity of odor, milk odor, and butter odor, all other sensory attributes that were the object of evaluation were not influenced by the addition of OEOs. 

In particular, the addition of OEOs increased odor intensity but influenced negatively milk and butter odor. These differences increased with OEOs’ concentration and followed the same trend commonly reported for cheeses produced with the addition of EOs [64,69]. With regard to overall satisfaction, intended as an overall rating of the cheeses expressed considering all attributes with their scores [78], cheeses produced with 100 μL/L of OEOs were particularly appreciated by the evaluators and reached a score higher than that registered for control cheeses. On the contrary, the overall satisfaction of Tuma cheeses enriched with 200 μL/L of OEO was lower than that of control products, confirming that the addition of high concentrations of EOs in processed foods can negatively alter their taste and aroma and, consequently, exceed acceptability for consumption [79].

### 3.9. Artificial Contamination Test

The results of plate counts carried out for all samples collected during the production of Tuma cheeses from milk artificially contaminated with dairy pathogens are reported in Table 5. 

Pasteurized ewe’s milk showed a TMM and coccus LAB count of approximately 3 Log CFU/mL, while dairy pathogenic bacteria were below the detection limit (<1 Log CFU/mL).

LAB and pathogens were found in inoculated milk used for control (CCP) and experimental products (ECP100 and ECP200) at 10^7^ and 10^4^ CFU/mL, respectively. As expected, both groups showed an increase of about one Log cycle after milk curdling. Cardamone et al. [80] observed a similar behavior in ewes’ milk inoculated with the same four pathogenic strains during the production of PDO Pecorino Siciliano cheese. Significant differences (*p* < 0.0001) were found for the levels of all dairy pathogenic bacteria between the control and experimental cheeses. However, although these microorganisms were found both in ECP100 and ECP200 products, their levels were three Log cycles lower than those registered for control prods. This observation is probably due to the limited contact time. Our results indicated a clear in vivo antibacterial activity of OEOs using Tuma cheese as a model cheese and confirmed previous observations of de Campos et al. [81]. These results confirmed those obtained by in vitro assay that showed very high antibacterial activity of OEOs against the main four dairy pathogenic bacteria, excluding any negative influence against LAB.

## 4. Conclusions

The addition of OEOs at 100 and 200 μL/L to milk did not affect the survival or growth of the two *Lc. lactis* starter cultures during cheese making. Experimental cheeses were characterized by the same ash, fat, and protein content in comparison to control products. OEOs’ addition significantly increased the antioxidant activity of Tuma cheeses and impacted cheese VOC profiles with carvacrol, suggesting a high carryover of the volatile fraction of EOs from milk to final cheeses. Sensory traits were not negatively affected by the addition of OEO, but the highest values of overall satisfaction were evidenced for the Tuma cheeses enriched with 100 μL/L of OEOs. The addition of OEO was not able to completely inhibit the growth of the main dairy pathogens in the final cheeses, but it reduced significantly their development in OEO-added cheeses. The results of this study clearly highlighted the positive role of OEOs to enlarge the Sicilian ewes’ milk-derived products portfolio, and may open new promising opportunities for the prevention of dairy pathogenic bacteria growth during cheese production.

## Figures and Tables

**Table 1:** Antibacterial activity of oregano essential oil.

Species	Strains	Inhibition (mm)	MIC (µL/mL)
Pro-technological			
*Lc. lactis*	NT1	-	n.d.
*Lc. lactis*	NT5	-	n.d.
Pathogenic			
*E. coli*	ATCC25922	42.5 ± 0.1	1.25
*L. monocytogenes*	ATCC19114	35.8 ± 0.2	2.50
*S.* Enteritidis	ATCC13076	42.0 ± 0.2	0.625
*St. aureus*	ATCC33862	38.2 ± 0.1	1.25

Results indicate the mean value of three independent assays. Abbreviations: MIC, minimum inhibitory concentration; *Lc.*, *Lactococcus*; *E.*, *Escherichia*; *L.*, *Listeria*; *S.*, *Salmonella*; *St.*, *Staphylococcus*; n.d., not determined. Symbols:—no inhibition found.

**Table 2:** Microbial load of raw and pasteurized ewe’s milk.

Microbial Counts	Samples		SEM	*p*-Value
	RM	PM		
TMM	6.02 ^a^	3.24 ^b^	0.45	<0.0001
Coccus LAB	5.71 ^a^	2.91 ^b^	0.45	<0.0001
Rod LAB	4.27 ^a^	1.49 ^b^	0.44	<0.0001
Enterococci	2.87 ^a^	<1 ^b^	0.46	<0.0001
Entericacteriaceae	3.03 ^a^	<1 ^b^	0.48	<0.0001
*E. coli*	2.79 ^a^	<1 ^b^	0.44	<0.0001
CPS	2.42 ^a^	<1 ^b^	0.38	<0.0001
*L. monocytogenes*	<1	<1	n.e.	n.e.
*Salmonella* spp.	<1	<1	n.e.	n.e.

Units are Log CFU/mL. Results indicate the mean values of six plate counts (carried out in triplicate for two independent products). Data within a row followed by different letters are significantly different according to Tukey’s test. Abbreviations: RM, raw milk; PM, pasteurized milk; SEM, standard error of the mean; TMM, total mesophilic microorganisms; LAB, lactic acid bacteria; *E.*, *Escherichia*; CPS, coagulase-positive staphylococci; *L.*, *Listeria*; n.e., not evaluated.

**Table 3:** Physicochemical and antioxidant traits of Tuma cheeses.

	Samples			SEM	*p*-Value
	CCP	ECPO100	ECPO200		
Cheese weight at 48 h, kg	2.93	3.52	3.61	2.244	0.2222
Cheese yield at 48 h, g/100 g	14.63	15.97	16.45	0.350	0.1206
Cheese yield at 48 h, g/100 g dry matter	8.65	9.02	9.34	0.208	0.1145
Dry matter (DM), %	59.16 ^a^	56.52 ^b^	56.79 ^b^	0.608	<0.0001
Ash, % DM	6.54	6.81	6.53	0.414	0.8531
Protein, % DM	43.34	42.39	43.24	1.413	0.5178
Fat, % DM	45.92	45.65	42.20	4.846	0.8041
pH	5.52	5.23	5.21	0.260	0.6878
Water activity, a_w_	0.971 ^b^	0.979 ^ab^	0.986 ^a^	0.015	0.0267
Hardness, N/mm^2^	0.451 ^a^	0.300 ^b^	0.347 ^ab^	0.041	0.0137
Lightness L*	83.27 ^b^	86.91 ^a^	86.12 ^a^	1.0988	0.0005
Redness a*	−3.551 ^b^	−3.161 ^a^	−3.078 ^a^	0.5096	0.0014
Yellowness b*	12.96 ^a^	12.48 ^ab^	11.94 ^b^	1.4333	0.0111
TEAC, mmol/kg DM	55.51 ^b^	68.26 ^b^	98.25 ^a^	4.763	0.0005
POV, mEq O_2_/kg fat	2.39 ^b^	3.14 ^a^	2.81 ^ab^	0.148	0.0089
TBARs, mg MDA/kg DM	0.066 ^c^	0.109 ^b^	0.172 ^a^	0.006	<0.0001

Results indicate mean values of determinations carried out in duplicate for each of the two independent cheese making processes. Abbreviations: CCP, control cheese product inoculated with the milk starter cultures (MSC); ECPO100, experimental cheese product inoculated with MSC + 100 μL/L of oregano essential oils (OEOs); ECPO200, experimental cheese product inoculated with MSC + 200 μL/L of OEOs; SEM, standard error of the mean. On the row: ^a^, ^b^ = *p* < 0.05.

**Table 4:** Volatile organic compounds emitted from Tuma cheeses.

Chemical Compounds	Samples			SEM	*p*-Value
	CCP	ECPO100	ECPO200		
Acids					
Acetic acid	6.93 ^a^	1.27 ^b^	1.09 ^b^	0.97	<0.0001
Butanoic acid	8.05 ^a^	2.65 ^b^	2.35 ^b^	0.96	<0.0001
Exanoinc acid	13.41 ^a^	2.94 ^b^	3.19 ^b^	1.76	<0.0001
2-hydroxy-4-methyl-Pentanoic acid	6.51 ^a^	1.98 ^b^	1.07 ^b^	0.86	<0.0001
Octanoic Acid	4.47 ^a^	1.54 ^b^	0.97 ^b^	0.57	0.001
Nonanoic acid	1.83 ^a^	0.30 ^b^	0.09 ^b^	0.29	<0.0001
Ketones					
2-pentanone	1.53 ^a^	0.10 ^b^	0.07 ^b^	0.25	<0.0001
3-hydroxy-2-butanone,	4.97 ^a^	0.80 ^b^	1.19 ^b^	0.70	0.001
2-heptanone	0.58 ^a^	0.010 ^b^	0.03 ^b^	0.09	<0.0001
2,3 octanedione	1.97 ^a^	0.66 ^b^	0.17 ^b^	0.28	0.000
3,5 octadien-2-one	0.39 ^a^	n.d. ^b^	n.d. ^b^	0.07	<0.0001
Alcohol					
3-Methyl-1-butanol	13.65 ^a^	4.76 ^b^	3.36 ^b^	1.70	0.001
1 pentanol	0.81 ^a^	0.19 ^b^	0.12 ^b^	0.11	<0.0001
2-butanol	2.64 ^a^	0.39 ^b^	0.24 ^b^	0.40	<0.0001
Octan-1-ol	2.79 ^a^	0.30 ^b^	0.50 ^b^	0.40	<0.0001
Hydrocarbons					
Hexane-2-methyl	1.46 ^a^	0.10 ^b^	n.d. ^c^	0.24	<0.0001
Heptane 2,4 dimenthyl	2.45 ^a^	0.90 ^b^	0.24 ^b^	0.34	<0.0001
Aldeyde					
4 heptenal	0.18	0.020	n.d.	0.04	0.169
Hexanal	13.03 ^a^	5.09 ^b^	4.34 ^b^	1.45	<0.0001
Heptanal	10.53 ^a^	3.98 ^b^	2.63 ^b^	1.29	0.001
Nonanal	1.82 ^a^	0.33 ^b^	0.09 ^b^	0.28	<0.0001
Monoterpenes					
α-Thujene	n.d. ^b^	0.20 ^a^	n.d. ^b^	0.03	<0.0001
α-Pinene	n.d. ^b^	0.06 ^a^	0.04 ^a^	0.01	0.000
Sabinene	n.d. ^c^	0.05 ^a^	0.01 ^b^	0.01	<0.0001
β-Pinene	n.d. ^c^	0.10 ^a^	0.01 ^b^	0.02	<0.0001
Myrcene	n.d. ^c^	0.90 ^a^	0.62 ^b^	0.13	<0.0001
α-Phellandrene	n.d. ^b^	0.11 ^a^	0.08 ^a^	0.02	<0.0001
α-Terpinene	n.d. ^b^	0.90 ^a^	0.81 ^a^	0.15	<0.0001
p-Cymene	n.d. ^b^	1.02 ^a^	0.89 ^a^	0.16	<0.0001
Limonene	n.d. ^b^	0.10 ^a^	0.11 ^a^	0.02	<0.0001
(Z)-β-Ocimene	n.d. ^b^	n.d. ^b^	0.21 ^a^	0.04	<0.0001
γ-Terpinene	n.d. ^b^	1.07 ^a^	0.99 ^a^	0.18	<0.0001
Monoterpenoids					
Linalool	n.d. ^b^	0.10 ^a^	0.10 ^a^	0.02	0.002
Thymol	n.d. ^c^	0.30 ^a^	0.20 ^b^	0.04	<0.0001
Carvacrol	n.d. ^c^	66.08 ^b^	73.09 ^a^	11.66	<0.0001
Camphor	n.d. ^b^	n.d. ^b^	0.10 ^a^	0.02	0.001
Terpinen-4-ol	n.d. ^b^	0.10 ^a^	n.d. ^b^	0.02	<0.0001
β-Bisabolene	n.d. ^b^	n.d. ^b^	0.20 ^a^	0.03	<0.0001
β-Caryophyllene	n.d. ^c^	0.60 ^b^	0.80 ^a^	0.12	<0.0001

Results are reported as relative peak areas (peak area of each compound/total area of identified VOC) × l00 and indicate the mean values ± standard deviation (S.D.) of four measurements (carried out in triplicate for two independent productions). Different superscript letters on the row indicate statistically significant differences according to Tukey’s test. The retention times and mean peak area values are reported in Table S1. Abbreviations: CCP, control cheese product inoculated with the milk starter cultures (MSC); ECPO100, experimental cheese product inoculated with MSC + 100 μL/L of oregano essential oils (OEOs); ECPO200, experimental cheese product inoculated with MSC + 200 μL/L of OEOs; n.d., not detectable.

**Table 5:** Microbial loads of samples collected during the artificial contamination test.

Samples	Microbial Counts					
	TMM	*Lc. lactis*	*E. coli*	*L. monocytogenes*	*S.* Enteritidis	*St. aureus*
Pasteurized milk	2.71	2.64	<1	<1	<1	<1
Inoculated milk						
CCP	7.09	7.01	3.99	3.91	4.02	3.88
ECPO100	7.13	7.06	3.86	3.82	3.97	3.80
ECPO200	7.08	7.09	3.90	3.90	4.05	3.99
SEM	0.06	0.05	0.06	0.05	0.05	0.06
*p* value	0.972	0.914	0.829	0.865	0.928	0.673
Curd						
CCP	7.96	7.95	4.90	4.82	4.79	5.01
ECPO100	8.03	7.87	4.91	4.73	4.82	4.94
ECPO200	8.01	7.92	4.84	4.66	4.76	5.05
SEM	0.05	0.04	0.05	0.06	0.04	0.04
*p* value	0.926	0.870	0.929	0.782	0.942	0.817
Cheese						
CCP	8.85	8.67	5.71 ^a^	5.31 ^a^	5.09 ^a^	5.66 ^a^
ECPO100	8.96	8.84	2.99 ^b^	2.55 ^b^	3.01 ^b^	3.11 ^b^
ECPO200	8.90	8.70	2.74 ^b^	2.23 ^b^	2.93 ^b^	3.01 ^b^
SEM	0.05	0.06	0.34	0.35	0.25	0.31
*p* value	0.857	0.727	<0.0001	<0.0001	<0.0001	<0.0001

Units are Log CFU/mL for milk samples and Log CFU/g for curds and cheeses. Results indicate the mean values of six plate counts (carried out in triplicate for two independent productions). Data within a column followed by the same letter are not significantly different according to Tukey’s test. Abbreviations: TMM, total mesophilic microorganisms; *Lc.*, *Lactococcus*; *E.*, *Escherichia*; *L.*, *Listeria*; *S.*, *Salmonella*; *St.*, *Staphylococcus*; CCP, control cheese product inoculated with the milk starter cultures (MSC); ECPO100, experimental cheese product inoculated with MSC + 100 μL/L of oregano essential oils (OEOs); ECPO200, experimental cheese product inoculated with MSC + 200 μL/L of OEOs.

## Data Availability

All data included in this study are available upon request by contacting the corresponding author.

## Supplementary Materials

The following supporting information can be downloaded at: https://www.mdpi.com/article/10.3390/antiox12061293/s1, Table S1. Volatile Organic Compounds determined by GC-MS in Tuma Cheeses.
