# Supplementary material for: Improvement of Fresh Ovine “Tuma” Cheese Quality Characteristics by Application of Oregano Essential Oils
Source: Antioxidants (Basel). 2023 Jun 17;12(6):1293. doi: 10.3390/antiox12061293 (PMC10294957; doi:10.3390/antiox12061293)
Supplement: Supplementary file 1 [file antioxidants-12-01293-s001.zip › antioxidants-2419763-supplementary.pdf]

antioxidants

MDPI

Supplemental Material

Improvement of Fresh Ovine "Tuma" Cheese Quality Characteristics by Application of Oregano Essential Oils

Giuliana Garofalo [1], Marialetizia Ponte [1], Carlo Greco [2], Marcella Barbera [3], Michele Massimo Mammano [2], Giancarlo Fascella [2], Giuseppe Greco [1,2], Giulia Salsi [2], Santo Orlando [1], Antonio Alfonzo [1], Antonino Di Grigoli [1], Daniela Piazzese [3], Adriana Bonanno [1], Luca Settanni [1] and Raimondo Gaglio [1,*]

[1] Department of Agricultural, Food and Forest Sciences, University of Palermo, 90128 Palermo, Italy; giuliana.garofalo01@unipa.it (G.G.); marialetizia.ponte@unipa.it (M.P.); giuseppe.greco@crea.gov.it (G.G.); santo.orlando@unipa.it (S.O.); antonio.alfonzo@unipa.it (A.A.); antonino.digrigoli@unipa.it (A.D.G.); adriana.bonanno@unipa.it (A.B.); settanni.luca@unipa.it (L.S.)

[2] Research Centre for Plant Protection and Certification, Council for Agricultural Research and Economics, 90011 Bagheria, Italy; carlo.greco@crea.gov.it (C.G.); massimo.mammano@crea.gov.it (M.M.M.); giancarlo.fascella@crea.gov.it (G.F.); giulia.salsi@crea.gov.it (G.S.)

[3] Department of Earth and Marine Sciences, University of Palermo, 90123 Palermo, Italy; marcella.barbera@unipa.it (M.B.); daniela.piazzese@unipa.it (D.P.)

* Correspondence: raimondo.gaglio@unipa.it

Table S1. Volatile Organic Compounds determined by GC-MS in Tuma Cheeses.

Identified Chemical Compounds	R.T.	Samples		
		CCP	ECPO100	ECPO200
Acids				
Acetic acid	14.0	5339032	978437.3	839761.2
Butanoic acid (butyric acid)	18.3	6201906	2041621	1802790
Hexanoic acid	22.1	10331374	2265044	2457650
2-hydroxy-4-methyl-Pentanoic acid	23.2	5015454	1525438	824352.7
Octanoic Acid	26.3	3443791	1186452	747310.4
Nonanoic acid	28.3	1409874	231126.9	69338.08
Ketones				
2-pentanone	15.3	1178747	77042.31	53929.62
3-hydroxy-2-butanone.	17.5	3829003	616338.5	916803.5
2-heptanone	19.9	446845.4	7704.231	23112.69
2.3 octanedione	21.9	1517733	508479.2	130971.9
3.5 octadien-2-one	23.4	300465	n.d.	n.d.
Alcohols				
3-Methyl-1-butanol (Isoamyl alcohol)	16.3	10516275	3667214	2588622
1 pentanol	17.1	624042.7	146380.4	92450.77
2-butanol	13.4	2033917	300465	184901.5
Octan-1-ol	23.9	2149480	231126.9	385211.5
Hydrocarbons				

Hexane-2-methyl	14.8	1124818	77042.31	n.d.
Heptane 2.4 dimenthyl	15.2	1887537	693380.8	184901.5
Aldehydes				
4 heptenal	19.9	138676.2	15408.46	n.d.
Hexanal	17.9	10038613	3921454	3343636
Heptanal	20.0	8112555	3066284	2026213
Nonanal	24.5	1402170	254239.6	69338.08
Monoterpenes				
α-Thujene	20.7	n.d.	154084.6	n.d.
α-Pinene	21.1	n.d.	46225.39	30816.92
Sabinene	24.2	n.d.	38521.15	7704.231
β-Pinene	23.1	n.d.	77042.31	7704.231
Myrcene	22.1	n.d.	693380.8	477662.3
α-Phellandrene	22.8	n.d.	84746.54	61633.85
α-Terpinene	23.0	n.d.	693380.8	624042.7
p-Cymene	22.9	n.d.	785831.6	685676.6
Limonene	23.2	n.d.	77042.31	84746.54
(Z)-β-Ocimene	22.8	n.d.	n.d.	161788.8
γ-Terpinene	23.7	n.d.	824352.7	762718.9
Monoterpenoids				
Linalool	24.6	n.d.	77042.31	77042.31
Thymol	28.9	n.d.	231126.9	154084.6
Carvacrol	29.2	n.d.	50909558	56310224
Camphor	25.9	n.d.	n.d.	77042.31
Terpinen-4-ol	26.7	n.d.	77042.31	n.d.
β-Bisabolene	33.9	n.d.	n.d.	154084.6
β-Caryophyllene	32.3	n.d.	462253.9	616338.5
Unknown compounds				
	25.5	594973.6	642571.5	597591.5
	31.1	312304.8	287320.4	293066.8
	35.7	207501.9	215802	192063.8

The results are expressed as peak area obtained from three replicates and include retention time (R.T.) of identified and unidentified compounds. Abbreviations: CCP, control cheese production inoculated with the Milk Starter Cultures (MSC); ECPO100, experimental cheese production inoculated with MSC + 100 μL/L of oregano essential oils (OEOs); ECPO200, experimental cheese production inocu-lated with MSC + 200 μL/L of OEOs; n.d., not detectable.
